# Quantification of Torque Teno Virus (TTV) in plasma and saliva of individuals with liver cirrhosis: a cross sectional study

**DOI:** 10.3389/fmed.2023.1184353

**Published:** 2023-06-22

**Authors:** Ana Clara Falabello de Luca, Gabriella Bueno Marinho, Juliana Bertoldi Franco, Jefferson da Rocha Tenório, Natália Silva Andrade, Alexandre Mendes Batista, Ana Carolina Mamana, Tânia Regina Tozetto-Mendoza, Mário Pérez Sayáns, Paulo Henrique Braz-Silva, Karem L. Ortega

**Affiliations:** ^1^Special Care Dentistry Centre (CAPE), Department of Stomatology, University of São Paulo, São Paulo, Brazil; ^2^Division of Dentistry, Clinics Hospital, University of São Paulo, School of Medicine, São Paulo, Brazil; ^3^Department of Pathology and Oral Diagnosis, Federal University of Rio de Janeiro, Rio de Janeiro, Brazil; ^4^Department of Dentistry, Federal University of Sergipe, Lagarto, Sergipe, Brazil; ^5^Laboratory of Virology, Institute of Tropical Medicine, School of Medicine, University of São Paulo, São Paulo, Brazil; ^6^Oral Medicine, Oral Surgery and Implantology Unit, MedOralRes Group, University of Santiago de Compostela, Health Research Institute of Santiago de Compostela (IDIS), Santiago de Compostela, Spain; ^7^Department of Stomatology, School of Dentistry, University of São Paulo, São Paulo, Brazil

**Keywords:** liver cirrhosis, cirrhosis-associated immune dysfunction, decompensate cirrhosis, Torque Teno virus, saliva, plasma

## Abstract

**Introduction:**

Torque teno virus (TTV) has been pointed as an endogenous marker of immune function, the objective of this study was to investigate the TTV viral load in plasma and saliva of cirrhotic individuals and correlate it with clinical characteristics.

**Methods:**

Blood, saliva, clinical data from records and laboratory tests were collected from 72 cirrhotic patients. Plasma and saliva were submitted to real-time polymerase chain reaction for quantification of TTV viral load.

**Results:**

The majority of the patients presented decompensated cirrhosis (59.7%) and 47.2% had alterations in the white blood series. TTV was identified in 28 specimens of plasma (38.8%) and in 67 specimens of saliva (93.0%), with median values of TTV copies/mL of 90.6 in plasma and 245.14 in saliva. All the patients who were positive for TTV in plasma were also positive in saliva, with both fluids having a moderately positive correlation for the presence of TTV. There was no correlation between TTV viral load, either in plasma or in saliva, and any of the variables studied.

**Conclusion:**

TTV is more frequently found and in greater amount in the saliva than in the plasma of cirrhotic patients. There was no correlation between TTV viral load and clinical parameters.

## 1. Introduction

Liver cirrhosis (LC) is characterised by replacement of the liver parenchyma with fibrosis, leading to two main clinical events: hepatic failure and portal hypertension (1). As a result of these changes, several complications and comorbidities occur, such as the cirrhosis-associated immune dysfunction (CAID) (2).

Liver plays a significant role in the immune-system homeostasis by contributing to the functioning of the reticuloendothelial system, mainly through the immune-surveillance and synthesis of soluble molecules regulating immune responses (3). As for CAID, one can observe anomalies in the adaptive and innate immune response, different degrees of immunodeficiency and systemic inflammation (4). In addition to the quantitative reduction, cirrhotic individuals present qualitative changes in the peripheral blood cells such as decreased phagocytic and chemotactic activity of neutrophils/monocytes, increased activation of T and B cells, and reduced cytotoxic activity of natural killer (NK) cells (5, 6). These alterations make the cirrhotic individual more susceptible to the development of several infections, mainly those of bacterial origin (2). When LC progresses into a state of decompensation, CAID becomes more pronounced and the immune response to bacteria becomes even more compromised (2).

Spontaneous bacterial peritonitis (SBP) is the most frequent infection in cirrhotic individuals, leading about 40 percent of the patients to death (7). Although some risk factors have been identified in the development of SBP, such as low amount of protein in ascitic fluid (<1 g/dL), low levels of 25-hydroxyvitamin D, serum bilirubin >4 mg/dL and prothrombin ≤45%, analyses of ascitic fluid and blood are not always performed in cirrhotic individuals at an adequate frequency due to the difficulty in collecting them (7).

Some recent studies have shown that Torque Teno Virus (TTV) is an endogenous marker of immune function, and consequently, the degree of immunosuppression in solid-organ-transplant patients (8). Both blood and saliva showed high viral loads of TTV in immunosuppressed individuals, in which assessment of saliva represents a non-invasive means of evaluating the immunological state of the patient (9).

TTV is a small, non-enveloped, single-stranded circular DNA virus with negative polarity (10) belonging to the genus of Alphatorquevirus of the Anelloviridae family (8). It is a ubiquitous and persistent virus, and as such, it can be considered part of the human virome. Immune-surveillance protects the body against the pathological effects of the virus (11) by restraining the viral replication, but not suppressing it. TTV is not known causes disease in healthy individuals and the viral load is increased in immunosuppressed patients with autoimmune or inflammatory diseases (10). Recent studies have identified the feasibility of using TTV to monitor the immune state of HIV patients (9), may be helpful for predicting the occurrence of severe nosocomial infections and mortality in patients with severe COVID-19 (12), predict graft outcomes in patients after solid organ transplantation and hematopoietic stem cell transplantation (13), predict clinical deterioration in intensive care unit patients (14), monitor immunosuppressed patients with rheumatoid arthritis who are on biological disease-modifying anti-rheumatic drugs (15) and indicate pulmonary function and severity of the disease in chronic obstructive pulmonary disease patients (16).

Therefore, the present study sought to investigate the TTV viral load in the plasma and saliva of cirrhotic individuals on the transplant waiting list and its relationship with the clinical picture of LC, in addition to examining whether saliva can be an efficient fluid for studying TTV in this group of patients.

## 2. Materials and methods

This cross-sectional study was approved by the Research Ethics Committee of the University of São Paulo School of Dentistry according to Declaration of Helsinki (protocol number 3895087) and conducted according to the Strengthening the Reporting of Observational Studies in Epidemiology (STROBE) recommendations for observational studies. All the patients were given verbal and written information about the research and agreed on participating in the study by signing an informed consent form.

The present study was conducted by using a convenience sample of 72 patients. Both adult male and female patients with LC who were on the liver transplant waiting list were selected for study.

Data on gender, age, diagnosis time of LC, Model of End-stage Liver Disease (MELD), complications and aetiology of cirrhosis, medications in use and presence of comorbidities were collected from the patients’ clinical records.

Saliva and blood were also collected, in which part of the collected blood was sent to the central laboratory for complete blood count and coagulation test. The remaining part of the collect blood and the saliva specimens were stored at -80°C until molecular analysis. The blood specimens were centrifuged and only plasma was stored (17).

Extraction of genetic material from the specimens was performed by using Loccus Extracta 32 equipment on a semi-automatic way according to the manufacturer’s instructions. Extractions were obtained from 200 μL of saliva and plasma. The extracted DNA was stored in a freezer at -20^o^ C until RT-PCR processing. A standard input of ~100 ng of DNA template was used per test.

The specimens were submitted to real-time polymerase chain reaction in which a set of primers and reagent probes specific for the untranslated region of the viral genome were used as described by Maggi et al. (18): primer forward 5-GTGCCGIAGGTGAGTTTA-3, primer reverse 5-AGCCCGGCCAGTCC-3 and probe 5-TCAAGGGGCAATTCGGGCT-3. Also, primers and B-actin gene probe were used for control of DNA quality. For reaction preparation, TaqMan^®^ Universal Master mix protocol (ThermoFisher Scientific, Warrington, United Kingdom) was used, according to the manufactory instructions. The cycling conditions were the following: initial activation step at 95°C for 10 min, followed by 40 cycles of denaturation at 95oC for 15 s and only one step of lengthening and extension at 60°C for 60 s by using a thermocycler (Light Cycler 96, Roche Diagnostics). A previously tested specimen was analysed in each reaction and quantified for TTV as a positive parameter, including contamination control of PCR reagents.

Positive specimens generated cycles threshold (CT), which were used as parameter for quantification of TTV viral load, whereas those presenting no amplification during the real time-PCR were considered negative.

Absolute quantification method was used to determine the TTV viral load. The synthetic standard curve used was based on the model described by Lima et al. (19). In this method, the CT of each specimen is compared to those presented by the points of the serial dilution curve with known concentrations of synthetic oligos target. The analytical sensitivity of the test is defined as the concentration of synthetic target that can be detected with a positivity rate of 95% (LoD ≥ 95%) by Probit analysis. The TTV DNA target was synthetized and HPLC purified by EXXTEND® (Oligos solutions, São Paulo, Brazil) as follows: 5′TTCGTAGCCCGGCCAGTCCCGTATAGCCCGAATTGCCCCTTGAATGCGTTAAACTCACCTTCGGCACCTGATA−3′, at initial concentration of 100 μM in TE (10 mM Tris, 0.1 mM EDTA, pH 8.0). In this study, the standard curve was performed with a ten-fold dilution series of synthetic oligos in nuclease free water. The low detection limit (LoD ≥ 95%) of test was 40 copies/mL (9, 29).

### 2.1. Statistical analysis

The resulting data were analysed by Statistical Package for the Social Science (SPSS^®^ for Windows, version 22.0, SPSS Inc., Chicago, IL, United States). Non-parametric data distribution was verified by the Kolmogorov Smirnov test. Spearman’s correlation coefficient was used to correlate the TTV viral load with laboratory blood data, such as the case of lymphocytic population. Bivariate analysis was performed by using Kruskal-Wallis and Mann Whitney tests for comparison of independent variables with the TTV viral load. All statistical analyses were performed at a significance level of 5%.

## 3. Results

The sample of 72 patients consisted predominantly of male individuals (*n* = 51; 70.8%), with median age of 57 years old (rank 26–71 years old). The median value of MELD was 15 (rank 7–50). The main aetiologies of LC were hepatitis C virus (31.4%), alcoholic cirrhosis (30%) and cryptogenic cirrhosis (8.6%). The inpatient follow-up ranged from 1 to 3 years and the majority of the patients presented with decompensated hepatic cirrhosis (59.7%). In Table 1, one can see the complications and comorbidities found.

**Table 1:** Clinical characteristics of cirrhotic patients included in the study (*n* = 72).

	*n* (%)
**Complications of cirrhosis**	
*Collateral Circulation (varices)*	
No	14 (19.4)
Yes	58 (80.6)
*Ascites*	
No	29 (40.3)
Yes	43 (59.7)
*Hepatic Encephalopathy*	
No	57 (79.2)
Yes	15 (20.8)
*Splenomegaly*	
No	12 (16.7)
Yes	60 (83.3)
*Spontaneous Bacterial Peritonitis*	
No	70 (97.2)
Yes	02 (2.8)
*Variceal Hemorrhage*	
No	54 (75)
Yes	18 (25)
**Comorbidities**	
Abnormal renal function^*^	
No	64 (88.9)
Yes	08 (11.1)
*Type II Diabetes Mellitus*	
No	54 (75.0)
Yes	18 (25.0)
*Arterial-coronary disease*	
No	64 (88.9)
Yes	08 (11.1)
*Systemic Arterial Hypertension*	
No	49 (68.1)
Yes	23 (31.9)
*Anemia*	
No	21 (29.1)
Yes	51 (70.8)

^*^Increase in circulating urea or creatinine.

The patients were categorized as being decompensated depending on the presence of clinically evident complications of portal hypertension (ascites, variceal haemorrhage, hepatic encephalopathy) or liver failure (jaundice).

The presence of abnormal renal function was characterised by an increase in circulating urea or creatinine, with no patient requiring hemodialysis, nor being diagnosed with hepato-renal syndrome.

Because of the presence of ascites, (19) (26.39%) patients had already undergone paracentesis, some more than twice (*n* = 8; 11.11%), but the majority had their condition controlled with medications.

In addition to comorbidities and complications of cirrhosis, it was also possible to observe that the majority of the patients presented changes in blood count and coagulation exams. Changes in the white blood series (decrease in the amount of leukocytes, neutrophils and lymphocytes) and thrombocytopenia were less frequent than changes in the red blood series (decrease in haemoglobin, red blood cells and haematocrit) (Table 2).

**Table 2:** Changes in blood cell components and coagulation in cirrhotic patients.

	*n* (%)
**Changes in the red blood series**	
No	21 (29.1)
Yes	51 (70.8)
**Changes in the white blood series**	
No	38 (52.7)
Yes	34 (47.2)
**Thrombocytopenia**	
No	46 (63.8)
Yes	26 (36.1)

The specimens of plasma and saliva from the 72 patients were submitted to analysis for identification and quantification of TTV. TTV was identified in (28) specimens of plasma (38.8%) and in 67 specimens of saliva (93.0%), in which the median values of TTV copies/mL were 90.6 and 245.14, respectively. All the patients who were positive for TTV in plasma were also positive in saliva. Only five patients had no TTV identified.

In parallel, information about amount of total leukocytes and some differentials (i.e., lymphocytes and neutrophils) was obtained in order to correlate the peripheral blood immune cells with TTV viral load (Table 3).

**Table 3:** Leukocytes and TTV viral load in the plasma and saliva.

Variables	Mean	S.D.	Min. Value	Max. Value	Percentiles		
					25	50	75
**Leukocytes** (*n* = 72)	4.07	1.79	0.93	9.38	2.66	3.70	4.95
**Neutrophils** (*n* = 72)	2.65	1.26	0.52	6.00	1.71	2.36	3.43
**Lymphocytes** (*n* = 72)	0.95	0.56	0.15	2.54	0.48	0.94	1.32
**TTV** (plasma) (*n* = 28)	124.25	147.12	0.73	540.22	9.87	90.61	160.58
**TTV** (saliva) (*n* = 67)	49542.83	387333.13	0.49	3172353.25	53.88	245.14	1335.52

Reference values: leukocytes: 3.9 to 10.2×103/μL; neutrophils: 1.5 to 7.7×103/μL; lymphocytes: 1.1 to 4.5× 103/μL.

The amount of viral copies of TTV found in the specimens of plasma and saliva was found to have a moderately positive correlation (Table 4).

**Table 4:** Correlation between leukocytes, MELD and TTV viral load in the plasma and saliva.

Variables	r (Spearman correlation coeficiente)	*p*-value
TTV in plasma vs. TTV in saliva	0.576	0.001
TTV in plasma vs. Leukocytes	0.231	0.289
TTV in saliva vs. Leukocytes	−0.161	0.223
TTV in plasma vs. Neutrophils	0.296	0.171
TTV in saliva vs. Neutrophils	−0.094	0.447
TTV in plasma vs. Lymphocytes	0.038	0.865
TTV in saliva vs. Lymphocytes	−0.209	0.112
TTV in plasma vs. MELD	0.305	0.115
TTV in saliva vs. MELD	0.016	0.899

MELD, model of end-stage liver disease; TTV, torque teno virus; vs.: versus.

The Spearman’s correlation coefficient was also used to assess the possibility of existing a correlation of TTV viral load in plasma and saliva with MELD and the amount of leukocytes, lymphocytes and neutrophils, but no positive results were found (Table 4).

The amount of TTV in plasma and saliva was compared to independent variables, such as presence of decompensated cirrhosis and cirrhosis aetiology, as shown in Tables 5, 6. Once more, it was not possible to find any association between TTV and the proposed variables.

**Table 5:** Comparison between amount of TTV in plasma and independent variables.

Variables	TTV in plasma			*p*
	*n*	Median	Interval	
*Decompensated cirrhosis*				
No	13	56.43	539.49	0.461^*^
Yes	15	113.07	435.74	
*Aetiology of cirrhosis*				
Alcoholic cirrhosis	06	233.81	483.79	
Cryptogenic cirrhosis	02	1.59	0.33	0.098^**^
Hepatitis C virus	10	63.26	226.89	
Other causes	08	117.58	435.74	

^*^ Mann–Whitney’s test; ^**^ Kruskal Wallis’ test.

**Table 6:** Comparison between amount of TTV in saliva and independent variables.

Variables	TTV in saliva			*p*
	*n*	Median	Interval	
*Decompensated cirrhosis*				
No	29	237.97	10808.51	0.081^*^
Yes	38	316.65	3172352.76	
*Aetiology of cirrhosis*				
Alcoholic cirrhosis	18	724.52	10802.26	
Cryptogenic cirrhosis	05	56.43	266.38	0.197^**^
Hepatitis C virus	23	243.09	35367.27	
Other causes	19	147.88	3172350.18	

^*^ Mann–Whitney’s test; ^**^ Kruskal Wallis’ test.

## 4. Discussion

TTV has been claimed as an immunocompetence biomarker for patients presenting some specific immunodeficiency conditions, but nothing is known on TTV and any specific state of immunodeficiency caused by cirrhosis.

As for CAID, immunodeficiency begins during the stage of compensated cirrhosis, progressively increasing to the decompensated stage and worsening in case of acute-on-chronic liver failure (ACLF) (2). The importance to identify a state of decompensation in cirrhotic patients relies on the existing correlation with the immune phenotype of the CAID. Low-grade systemic inflammatory phenotype is present in compensated patients or decompensated ones with no organ failure, whereas high-grade systemic inflammatory phenotype is present in patients with ACLF (2). These two phenotypes represent two extremes of a spectrum of immune alterations. Because the intensity of CAID is directly correlated with severity of hepatic insufficiency, bacterial translocation and failure of other organs, it is important to characterise it (2).

In the present study, we found several indications that the studied population could present CAID, and therefore, be at higher risk of developing SBP. In addition to two patients who had already presented SBP before, more than a half of the patients (59.7%) had ascites, which is one of the most important signs of cirrhotic decompensation, being directly associated with an increased susceptibility to bacterial infection (6). Moreover, changes in white blood series were observed in almost half of the patients studied. With such a clinical picture, it was already expected that the immune response in this group of patients could be compromised.

Evaluating the efficiency of the immune system in cirrhotic patients has been considered by some authors as an important tool for prediction and control of CAID. The development of biomarkers of immune response can be useful in the risk stratification of patients by identifying those who can benefit from immune-therapeutic approaches and assessing the results of therapeutic interventions (20). However, the methodologies which could be used for this objective are complex, expensive and poorly studied (21).

TTV has recently emerged as a good candidate for staging the immunological condition of the immunosuppressed patients (8, 22). Both blood/plasma and saliva have already been shown to be useful in the quantification of TTV and its correlation with immunosuppression states in transplanted as well as in HIV-immunosuppressed patients (8, 9).

In the present study, TTV was more frequently detected and in higher viral load in saliva than in plasma. This finding was also reported elsewhere, both in healthy patients (23, 24) and immunosuppressed ones (25), making the authors propose that oral cavity is a possible site of replication and source of TTV transmission (26). In addition, this finding seems to be promising regarding the possibility of using saliva for identification and monitoring of the states of immunosuppression.

It is known that the shedding dynamics of some viruses in the saliva can vary significantly as a result of several clinical characteristics (27). Therefore, it is important to emphasise that our findings identified a certain positive correlation, despite being moderate, between TTV viral load in plasma and saliva. This correlation has already been more consistently identified by other studies (25).

In our patients, we found higher mean values of viral load in plasma (124.24 copies/mL) than those previously described in healthy patients (25 copies/mL) (28), but very inferior to those identified in patients with onco-haematological diseases (398 copies/mL) (25), chronic kidney disease (7,064.19 copies/mL) (29) and HIV (199,526 copies/mL) (30).

In saliva, despite the extremely high mean of TTV viral load (49,542.83 copies/mL), one can observe a high standard deviation in which the great majority of the patients (percentile 75) also had much lower values (1,335.52 copies/mL) than those found in immunosuppressed patients with onco-haematological diseases (125,983 copies/mL) (25), chronic kidney disease (2,562.58 copies/mL) (29) and HIV (501,187 copies/mL) (9).

These viral load results lead us to suppose the existence of immunosuppression in the cirrhotic patients evaluated, but the severity is not equivalent to that of other groups of immunosuppressed patients. On the other hand, the low positivity and significantly lower viral load of TTV found in the plasma of these patients may be linked to the increased serum concentrations of nitric oxide (NO), which is associated with the haemodynamic alterations induced by portal hypertension (31). NO has been known as an agent with potent antiviral activity and a complex role in the immunological host responses to viral infections, depending on its concentration and the type of virus (32, 33).

In parallel, it was not possible to determine any correlation between TTV viral loads (i.e., plasma and saliva) and any clinical characteristic of cirrhotic patients (i.e., amount of leukocytes, MELD, presence of decompensation and aetiology of cirrhosis). The lack of correlation between TTV viremia and absolute count of lymphocytes was already expected and been reported by earlier studies. In fact, the correlation between leukocytes and TTV occurs with certain subsets of lymphocytes (TCD4+) and more intensely in function of these cells (34, 35). In any way, at least 75% of our patients had counts of leukocytes within the normality, which might explain the fact that no correlation was found.

Nevertheless, it was expected to find a correlation with the stage of decompensation in the patients. It is probable that this did not occur because the majority of the patients, despite the decompensation, were in relatively favourable clinical conditions as they presented no extra-hepatic organ failure, which characterizes the presence of ACLF (36).

Hepatic cirrhosis, like any other chronic systemic disease, has shades as the patients can undergo different stages of severity of the disease. Therefore, in order to evaluate the population of cirrhotic patients more consistently, it would be necessary to increase the number of patients with more severe conditions. We believe that this was one of the limitations of our study (small sample). Another limitation was the study design (i.e., cross-sectional). Because this population had not yet been studied in relation to the presence and quantification of TTV, it is important to evaluate this group of patients over time, following their clinical evolution and comparing it to the TTV viral load in plasma and saliva.

Nevertheless, this is the first study investigating the efficiency of the immune system of cirrhotic patients and its correlation with TTV viral load as a biomarker. Further studies are necessary to better understand the function of TTV in the context of the immunosuppression caused by cirrhosis (37).

In the present work, TTV was more frequently identified in the saliva than in the plasma of cirrhotic patients, with higher viral load in the former. Nevertheless, it was not possible to determine a correlation between TTV viral load and degree of immunosuppression on the basis of the white blood cell count in cirrhotic patients.

## Data availability statement

The raw data supporting the conclusions of this article will be made available by the authors, without undue reservation.

## Ethics statement

The studies involving human participants were reviewed and approved by Research Ethics Committee of the University of São Paulo School of Dentistry (protocol number 3895087). The patients/participants provided their written informed consent to participate in this study.

## Author contributions

PB-S and KO: conceptualization. AF, GM, JF, JT, AB, and AM: methodology. TT-M and MP: validation. KO, PB-S, TT-M, and MP: formal analysis. AF, GM, JF, JT, AB, AM, and NA: investigation. TT-M and PB-S: resources and writing—review and editing. AF, GM, NA: data curation. AF, KO, and MP: writing original draft preparation. JT: visualization. KO and PB-S: supervision. AB and AM: project administration. PB-S: funding acquisition. All authors contributed to the article and approved the submitted version.

## Funding

The work was supported by Fundação de Amparo à Pesquisa do Estado de São Paulo–FAPESP (Grants 2017/18938-6; 2021/07490-0; 2021/00708-0) and Pró-Reitoria de Pesquisa da Universidade de São Paulo (Grant 2021.1.10424.1.9).

## Conflict of interest

The authors declare that the research was conducted in the absence of any commercial or financial relationships that could be construed as a potential conflict of interest.

## Publisher’s note

All claims expressed in this article are solely those of the authors and do not necessarily represent those of their affiliated organizations, or those of the publisher, the editors and the reviewers. Any product that may be evaluated in this article, or claim that may be made by its manufacturer, is not guaranteed or endorsed by the publisher.
